# Cellular Regulation of Macropinocytosis

**DOI:** 10.3390/ijms25136963

**Published:** 2024-06-26

**Authors:** Yumeng Wu, Xiao Hu, Zhixiao Wei, Qiong Lin

**Affiliations:** School of Medicine, Jiangsu University, Zhenjiang 212013, China; 2212213095@stmail.ujs.edu.cn (Y.W.); 2212213071@stmail.ujs.edu.cn (X.H.); 2212213070@stmail.ujs.edu.cn (Z.W.)

**Keywords:** macrropinocytosis, small GTPases, phosphatidylinositol, macropinosome, methuosis, cancer

## Abstract

Interest in macropinocytosis has risen in recent years owing to its function in tumorigenesis, immune reaction, and viral infection. Cancer cells utilize macropinocytosis to acquire nutrients to support their uncontrolled proliferation and energy consumption. Macropinocytosis, a highly dynamic endocytic and vesicular process, is regulated by a series of cellular signaling pathways. The activation of small GTPases in conjunction with phosphoinositide signaling pivotally regulates the process of macropinocytosis. In this review, we summarize important findings about the regulation of macropinocytosis and provide information to increase our understanding of the regulatory mechanism underlying it.

## 1. Introduction

**Figure 1:**
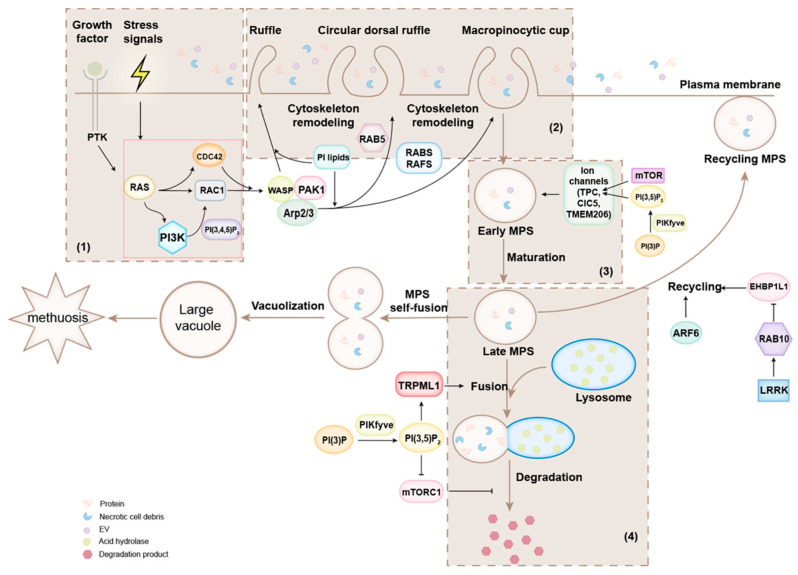
Cellular process and regulation of macropinocytosis. As shown in the colored areas, macropinocytic process is divided into 4 stages: (**1**) initiation and activation of macropinocytosis; (**2**) formation of macropinosomes; (**3**) maturation of macropinosomes; and (**4**) degradation and recycling of macropinosomes. Macropinocytosis is activated by a number of extracellular signals, such as growth factors and stress factors like amino acid deficiency and hypoxia. These signals either directly or indirectly activate RAS/RAC1/PI3K signaling to initiate plasma membrane ruffle formation. The formation of macropinosomes is involved in actin cytoskeleton remodeling followed by activation of RAS/RAC1/PI3K signaling. Multiple RAB proteins, such as RAB5, RAB20, RAB21, and RAB34, and ARF proteins, such as ARF6 and ARF1, also participate in the process along with the RAS/RAC1/PI3K proteins. The maturation of macropinosomes is osmatically processed by ion channels, such as TPC for Na^+^, 2Cl^−^ /H^+^ exchanger ClC5, and Cl^−^ channel TMEM206, for adjusting the volume of macropinosomes. The matured macropinosomes are transported to and fused with lysosomes for degradation. The fusion of matured macropinosomes with lysosomes is promoted by TRPML1, and degradation of macropinosomes is inhibited by mTORC1. Some macropinosomes can be recycled back to plasma membrane, and the recycling process is regulated by EHBP1L1 and ARF6. When macropinocytosis is over-activated and impaired, excessive macropinosomes can undergo vacuolization by self-fusion and form large dysfunctional vacuoles, leading to the non-apoptotic cell death called methuosis. The RAS/RAC1/PI3K proteins are the core regulatory proteins in macropinocytosis and their main function is generating plasma membrane ruffles, which is the essential cellular process for formation of macropinosomes. The major regulatory pathways in each of the process stages are labelled in the figure and described in the text. PI lipids stands for phsphatidylinositol lipids, including PI(3,4)P2, PI(4,5)P2, and PI(3,4,5)P3. PI(3,4)P2, phosphatidylinositol 3,4-bisphosphate; PI(4,5)P2, phosphatidylinositol 4,5-bisphosphate; PI(3,4,5)P3, phosphatidylinositol-3,4,5-trisphosphate; EV, extracellular vesicle; MPS, macropinosome; RTK, receptor tyrosine kinase.

Macropinocytosis is a type of non-selective endocytosis in which extracellular fluid is taken into the cell through the internalization of the plasma membrane (Figure 1) [1,2]. Macropinocytosis is characterized by initiating plasma membrane ruffles on the cell surface where it takes place and subsequently engulfing the extracellular fluid by the formation of giant (0.2–5 µm in diameter) and irregularly shaped endocytic vacuoles, named macropinosomes [3]. The extracellular fluid taken by macropinosomes contains proteins, necrotic cell debris, cell surface receptors, or/and extracellular vesicles (EVs) and provides sources of nutrition for the cell [4]. The contents of macropinosomes are generally transported to lysosomes for degradation.

It has been shown that growth factors, oxidative stress, and starvation are able to trigger macropinocytosis (Figure 1) [4]. Cancer cells under nutritional stress utilize macropinocytosis to obtain nutrients for proliferation and metabolism [5]. Particularly, cancer cells expressing oncogenic RAS mutants (e.g., K-RAS and H-RAS) exhibit high macropinocytosis activity and may develop into methuosis, a type of cell death linked to macropinocytosis [6]. Dendritic cells (DCs) and macrophages utilize macropinocytosis to acquire and present antigens [7]. Viruses and bacteria sometimes invade cells or facilitate uptake by host cells through macropinocytosis [8].

As shown in Figure 1, the general processes of macropinocytosis include four steps: (a) the initiation and activation of macropinocytosis; (b) formation of plasma membrane ruffles and macropinocytic cups; (c) maturation of macropinosomes; and (d) fusion of macropinosomes with lysosomes for the degradation or recycling of macropinosomes. The signaling for the regulation of macropinocytosis normally starts with the activation of RAS protein by macropinocytic stimulators, which in turn initiate signaling cascades involving RAC1 and phosphatidylinositol kinases and their downstream effectors or product that lead to actin cytoskeleton remodeling for plasma membrane ruffle and macropinocytic cup formation [1,9,10,11]. The macropinocytic cups wrap the extracellular fluid and form macropinosomes [1,9]. Macropinosomes are further matured by regulation of the small GTPases RAC1 and CDC42 as well as phosphatidylinositol lipids, and matured macropinosomes undergo recycling or degradation processes.

In this review, we focus on the regulatory process of macropinocytosis and hopefully increase our understanding of the regulatory mechanism underlying the macropinocytic process.

## 2. Signaling Pathways for Initiation and Activation of Macropinocytosis

The small GTPase RAC1 was the first protein found to initiate plasma membrane ruffles and macropinocytosis [12]. Subsequently, numerous studies connected plasma membrane ruffles to the initiation of macropinocytosis and identified RAC1 as a key regulatory protein in the initiation of macropinocytosis and the formation of macropinosomes. The pro-oncogenic protein RAS, a small GTPase upstream of RAC1 and PI-3 kinase, has been identified as an essential regulatory protein for macropinocytosis in the nutrition acquisition of cancer cells [13]. The RAS/RAC/PI3K signaling axis is currently considered as the pivotal regulatory pathway for generating plasma membrane ruffles, initiating macropinocytosis, and forming macropinosomes.

It has been observed that amino acid starvation activates macropinocytosis in cancer cells through the AMPK, YAP, or EGFR signaling pathways [14,15,16]. It seems that all these signaling pathways point to the activation of RAS and/or RAC. In addition to EGFR, multiple tyrosine kinase receptors, such as hepatic growth factor receptor (c-Met), platelet-derived growth factor receptor (PDGFR), colony-stimulating factor-1 receptor (CSF-1R), and AXL, also activate macropinocytosis [17,18,19,20]. Macropinocytosis is also activated by Wnt signaling [21,22] and immune stimulating signaling, such as interleukin signaling [23]. These signaling pathways activate downstream K-RAS or H-RAS and RAC, leading to the remodeling of the actin cytoskeleton and the formation of membrane ruffles. Macropinocytosis is intensely reliant on the phosphoinositide 3-kinase (PI3K) isoform PI3Kβ [24]. It has been demonstrated that macropinocytosis is impaired by PI3K inhibitors, such as GDC-0941, ZSTK474, wortmannin, and LY294002 [10,20,25].

In immune cells, such as DCs and macrophages, macropinocytosis may constitutively takes place [23,26]. In comparison, macropinosomes from constitutive macropinocytosis, which is dependent on calcium-sensitive receptors (CaSRs), tend to be smaller than those induced by growth factors [27]. CaSR, a G protein-coupled receptor (GPCR) [28], responds to extracellular calcium and initiates a signaling cascade that promotes the constitutive expansion of membrane ruffles and the formation of macropinosomes [27].

## 3. Regulation of Macropinosomal Formation

The initiation of macropinocytosis commences with the formation of membrane ruffles. There are two types of plasma membrane ruffles: peripheral ruffles (PRs) and circular dorsal ruffles (CDRs) [29,30], both of which are able to develop into macropinosomes. During the process of forming plasma membrane ruffles, the remodeling of the actin cytoskeleton is a critical step, as evidenced by the fact that actin polymerization disruptive reagents (e.g., cytochalasin D) inhibit the formation of macropinosomes [31]. The RHO small GTPase family member RAC plays the most important role in the remodeling of the cytoskeleton for membrane ruffles. The plasma membrane inositol phospholipids are key players in actin cytoskeleton remodeling, activating RAC and recruiting actin cytoskeletal regulatory proteins. Phosphatidylinositol-3,4,5-trisphosphate (PI(3,4,5)P3) is able to recruit the guanine nucleotide exchange factors T-lymphoma invasion and metastasis 1 (TIAM1) and dedicator of cytokinesis 2 (DOCK2) to the plasma membrane which subsequently activates RAC [32]. Phosphatidylinositol 4,5-bisphosphate (PI(4,5)P2), which activates the suppressor of cAMP receptor (SCAR)/WASP family verprolin homologous (WAVE) complex, and the RAC effector Wiskott–Aldrich syndrome protein (WASP), which interacts with PI(4,5)P2, actin, and actin-related proteins 2/3(Arp2/3), coordinate the assembly of the Arp2/3 complex that promotes actin polymerization [28], leading to the rearrangement of the actin cytoskeleton for the formation of ruffles [2].

The formation of CDRs is different from that of PRs, as it requires extra molecular machines, such as RAB5 GTPase, for the formation of circular ruffles in addition to RAS and RAC [30]. It was observed that the constitutively active mutant of RAS or RAC1 alone was not enough to induce CDRs [2]. The overexpression of the inactive mutant of RAB5, RAB5-S34N, inhibits circular ruffle formation in PDGF-stimulated mouse embryonic fibroblast (MEF) cells [33]. Increasing the expression of RAB5 together with the active form of RAS enhances the formation of circular ruffles [33,34]. Upon the formation of circular ruffles, they are closed by ruffle-like motility, sequestering large amounts of extracellular fluid in macropinocytic cups, and form macropinosomes when these ruffles fuse with the plasma membrane and encapsulate large amounts of extracellular fluid.

Multiple small GTPases, including RHO family GTPases, ARF GTPases, RAB GTPases, and inositol phospholipids, play pivotal roles in the process of macropinocytic cup closure and macropinosomal formation. RAC1 is one of the most important small GTPases for the regulation of membrane ruffles and macropinosomal formation in a wide range of cell types, including DCs, macrophages, fibroblasts, and epithelial cells [10]. RAC1 activates actin nucleation by interacting with downstream effectors such as PAK1 and WASP for the remodeling of the actin cytoskeleton and promotes the formation of ruffles and macropinocytic cups in the plasma membrane [28,35]. RAC1 is inactivated when macropinocytic cups are occluded to form macropinosomes [36]. RHOG is homologous to RAC1 and functions as an upstream activator of RAC1 [37,38]. It was observed that the RHOG-mediated activation of RAC1 is crucial for the formation of the macropinocytic cup in fibroblasts and A431 cells [10,37,39]. RHOC is transiently activated at the circular ruffles prior to the closure of macropinocytic cups for the formation of macropinosomes [40]. RHOA activation was observed following the closure of macropinocytic cups [41]. The ARF family members ARF6 and ARF1 also play important roles in macropinocytosis. Upon RAC1 activation, ARF6 elicits the accumulation of ARNO (ARF-guanine nucleotide exchange factor GEF) for the activation of ARF1 and actin cytoskeletal remodeling [42]. Furthermore, ARF6 activates phospholipase D1, which is required for macropinosomal formation [10]. During macropinosomal formation, RAB5a is activated and recruited to the macropinocytic site. By interacting with its downstream effector, Rabankyrin-5, RAB5 regulates actin cytoskeletal remodeling and promotes membrane ruffles and macropinosomal formation [42]. There are studies claiming that RAB34 engages in membrane ruffling and regulates the formation of macropinosomes [43,44], but this is generally contingent on the activity of other small GTPases, such as RAS [45] and RAC1 [43].

Inositol phospholipids, including PI(4,5)P2, PI(3,4,5)P3, and phosphatidylinositol 3,4-bisphosphate (PI(3,4)P2), are involved in macropinosomal formation [42]. A marked increase in PI(4,5)P2 levels has been reported when plasma membrane ruffling occurs [46]. PI(4,5)P2 recruits various actin-binding proteins to the plasma membrane and initiates actin polymerization and reorganization, which are necessary for membrane ruffling. The small GTPase RAC1 or ARF6 promotes the production of PI(4,5)P2 by activating PI4P5K, the enzyme that phosphorylates phosphatidylinositol 4-phosphate (PI4P) to form PI(4,5)P2 [47]. Upon membrane ruffle closure to form macropinocytic cups, the level of PI(4,5)P2 at the ruffles decreases along with a significant increase in the level of PI(3,4,5)P3 [3,48]. PI(3,4,5)P3 binds to the PH domain of multiple actin remodeling proteins that activate their downstream signaling effectors for actin cytoskeletal reconstruction [10]. Subsequent to macropinocytic cup closure, PI(3,4,5)P3 is dephosphorylated to form PI(3,4)P2 by SHIP 5-phosphatase [46].

Different from other forms of endocytosed vesicles, macropinosomes have no apparent cortical structure and are considerably larger. Since macropinosomes can be as large as 5 μm in diameter, they can easily be identified by using liquid phase markers, such as lucifer yellow (LY), horseradish peroxidase, or dextran [29,49].

## 4. Regulation of Maturation and Transport of Macropinosomes

Macropinosomes shrink as they mature, allowing the cell to resolve massive amounts of internalized material by macropinocytosis [50]. A loss of fluid in macropinosomes is required to maintain cell size and ionic composition. The contraction of macropinosomes is regulated by the two-pore channel (TPC)-mediated Na+ efflux and the Cl^−^ channel, namely ClC-5, together with the pH-regulated ASOR/TMEM206 channel-mediated Cl^−^ efflux [50]. TPC is a transmembrane channel dedicated to Na+ transport [50] and induces cell water loss through osmosis [42]. Thus, TPC is capable of reducing the hydrostatic pressure of macropinosomes [42]. TPC is regulated by PI(3,5)P2, an inositol phospholipid product of the phosphoinositide kinase, FYVE-type zinc finger containing (PIKfyve) [51], and mTOR kinase [52,53]. PI(3,5)P2 is a potent activator of TPC and promotes the process of macropinocytosis; thus, PIKfyve kinase activity directly controls macropinocytosis [51]. mTOR phosphorylates TPC when the cell has adequate nutrition and the phosphorylated TPC is inactivated; thus, macropinocytosis is inhibited [52,53]. When the cell is in starvation condition, mTOR is inactive, and the TPC channel is open, the macropinocytotic process is activated. Water loss due to TPC not only causes a decrease in the size of macropinosomes but also induces the formation of tubular extensions of macropinosomes [3]. The tubules detach from the macropinosomes by membrane splitting and are subsequently sorted and transported to specific intracellular compartments in a microtubule-dependent transport mode [54].

RAB GTPases are actively involved in macropinosomal transport [55]. As a molecular switch, RAB GTPases interact with downstream effectors to regulate various aspects during macropinosomal trafficking [56]. RAB5 is known for the regulation of the early stage of macropinocytosis and co-localization with early macropinosomes. It seems that the dissociation of RAB5 from early macropinosomes is a key step for the maturation and transport of macropinosomes. One of the determination processes is the RAB5/RAB7 transition [10]; i.e., RAB5 is replaced with RAB7 on macropinosomes for their maturation and lysosomal transport. It has been shown that active RAB7 interacts with amyotrophic lateral sclerosis 2 (ALS2), an activator for RAB5, dissociates ALS2 from RAB5, and inactivates RAB5 [57], thus replacing RAB5 on macropinosomes [57]. In phagocytic cells, the recycling of macropinosomes is specifically regulated by the RAB10/ leucine-rich repeat kinase (LRRK) signaling axis [58]. The RAB10 effector EH domain binding protein 1-like 1 (EHBP1L1) mediates early macropinosomal recycling and the phosphorylation of RAB10 by LRRK blocking the recycling effect of EHBP1L1. This phosphorylation enables early macropinosomes to avoid recycling back to the plasma membrane and the further maturation of macropinosomes, which is crucial for macropinosome-mediated immune responses [58]. In addition, RAB20 and RAB21 may be involved in the maturation of macropinosomes [59,60]. It was observed that RAB21 and RAB20 were recruited to the membrane of early macropinosomes. While RAB21 dissociated from Lamp1-positive macropinosomes, RAB20 remained on the RAB7 and Lamp1-positive macropinosomes, suggesting that RAB20 may directly participate in the maturation of macropinosomes [59,60]. However, the exact role of RAB20/RAB21 in the maturation of macropinosomes is still not clear.

Nevertheless, as RAB GTPases regulate processes in the transport of various types of membrane vesicles, how to sort out the specific roles of RAB GTPases in macropinosomal maturation and transport is a big challenge for future studies.

## 5. Regulation of Degradation Process of Macropinocytosis

The matured macropinosomes undergo either recycling or degradation. It has also been proposed that the transport destination of macropinosomes is determined by the context of signaling pathways in macropinocytosis [61]. Macropinosomes derived from RAS- or RAC-activated macropinocytosis are primarily transported to lysosomes, whereas ones from ARF6-activated macropinocytosis are recycled back to the cell surface [62]. The proportion of those undergoing recycling versus lysosomal degradation may vary among cell types, cargos, and stages of macropinocytosis [63].

Similar to endosomal trafficking, macropinosomal trafficking requires vesicle acidification. The disruption of vesicle or lysosomal acidification inhibits macropinocytosis and macropinosomal transport. Hydroxychloroquine (HCQ), a lysosomal inhibitor, inhibits macropinocytosis by interrupting lysosomal acidification [64]. The vesicular H(+)-ATPase (V-H(+)-ATPase), as a proton pump, is essential for maintaining vesicle acidification [65]. The V-H(+)-ATPase inhibitor bafilomycin A1 inhibits the vacuolization of macropinosomes [66] and blocks macropinosome–lysosome fusion during macropinocytosis [67]. It was observed that bafilomycin A1 blocks the formation of plasma membrane ruffling in macropinocytosis, suggesting that V-H(+)-ATPase may reside in the plasma membrane and regulate extracellular pH levels, which affect the formation of plasma membrane ruffles [31]. In fact, V-H(+)-ATPase is localized at the plasma membrane of the K-RAS mutant PDAC cells [68,69]. In addition, the downregulation of PIKfyve prevents the fusion of late endosomes or lysosomes with macropinosomes containing EGFR, and the effect of PIKfyve may be mediated by its downstream effector transient receptor potential mucolipin 1 (TRPML1), which was observed to modulate the size of vacuoles [42,52]. It has been shown that PI(3,5)P2, the product of PIKfyve, regulates the lysosomal Ca^2+^ channel which is crucial for lysosomal membrane fusion [3]. PI(3,5)P2 mediates macropinosome–lysosome fusion through the activation of TRPML1 [70]. Furthermore, when TRPML1 is inactivated upon the low activity of PIKfyve, the activation of mTORC1 is induced and the catabolism of macropinosomes is restrained [71]. These data indicate that PIKfyve and TRPML1 play important roles in macropinosome–lysosome fusion and the degradation of macropinosomes. In addition, it was observed that septins associate with matured macropinosomes in a PI(3,5)P2-dependent manner and facilitate the transportation and fusion of macropinosomes with lysosomes [50]. The knockdown of septin2 causes the accumulation of macropinosomes and formation of large vacuoles. This study indicates that septin2 is a key regulator in macropinosome maturation and lysosomal degradation. However, the molecular mechanism underlying the promotion of septins of macropinosomal trafficking to and fusion with lysosomes currently remains unknown.

## 6. Vacuolization of Macropinosomes and Methuosis

Macropinosomes normally undergo a maturation process by which they are either degraded by late endosomal/lysosomal processes or recycled back to the plasma membrane for supplying nutrients and maintaining cell membrane balance [29]. However, when abnormally active macropinocytosis produces excessive macropinosomes that are unable to fuse with lysosomes and recycle back to the plasma membrane, the macropinosomes fuse with each other and form huge vacuoles in the cell that have no markers of autophagosomes (LC3), early endosomes (RAB5 and EEA1), or recycling endosomes (RAB11) [72] but have markers of late endosomes and lysosomes (RAB7 GTPase and membrane glycoprotein Lamp-1). The vacuoles lack hydrolases, are not acidic, and have no late endosomal function [73]. This vacuolization results in cell death, which is called methuosis. 

Methuosis was initially observed in the glioblastoma cell overexpression of an oncogenic RAS mutant [74]. Methuosis is a novel form of non-apoptotic cell death characterized by the accumulation of large vacuoles derived from macropinosomes that eventually lead to membrane rupture, cell lysis, and death [70]. This unique cell death with morphological features is distinct from both apoptosis and other non-apoptotic cell death forms, such as autophagy and necrosis [75]. Methuosis usually occurs in tumor cells, such as non-small cell lung cancer cells (A549), prostate cancer cells (DU145), gastric cancer cell lines (HGC-27), and HeLa cells, but sometimes also occurs in normal cells (e.g., primary chicken cardiomyocytes and fibroblasts) with overloaded vesicles [76].

Studies have shown that methuosis has no connection with RAS/MAPK signaling [77,78]. Moreover, the process of methuosis does not depend on the activity of class I PI3K, the RHOA GTPases, or the RALA GTPase but on the downstream effectors of the oncogenic RAS [73]. RAC is the downstream signaling molecule of RAS that drives methuosis [79]. The unrestricted activation of RAC1 by RAS (G12V) stimulates the formation of plasma membrane ruffles and macropinosomes. At the same time, activated RAC1 interacts with GIT1, a GAP protein of ARF6, and causes the inactivation of ARF6 [78]. As ARF6 plays a role in the promotion of the recycling of macropinosomes to the plasma membrane [78], the inactivation of ARF6 by RAC1 reduces the recycling of macropinosomes. Consequently, excessive macropinosomes accumulate within the cell, fuse with each other, and form extremely large vacuoles. These large vacuoles are not functional and degradable, ultimately leading to methuosis [42].

In recent years, several methuosis inducers have been identified. All of these inducers have shown killing effects on cancer cells. This has increased interest in the application of methuosis inducers for cancer therapy. The prototype compounds 3-(5-methoxy-2-methyl-1H-indol-3-yl-3-yl)-1-(4-pyridyl)-2-propen-1-one (abbreviated as MOPPEP) and chalcone 3-(2-methyl-1H indol-3-yl)-1-(4-pyridinyl)-2-propen-1-one (abbreviated as MIPP) were reported to induce methuosis in cancer cell lines at low micromolar concentrations [75,80]. A recent study identified PIKfyve as the specific target protein of MOMIPP in the induction of methuosis [81]. PI(3,5)P2, the product of PIKfyve, is known to play a key role in late-stage macropinosomal trafficking and may mediate the effect of MOMIPP on cellular methuosis [82,83]. However, excessive vacuolization and methuosis induced by inducer chemicals seem to be independent of the RAS-RAC1 signaling pathway [76].

## 7. Macropinocytosis and Cancer Therapy

Three anticancer strategies based on macropinocytosis have been developed, including delivering chemo/targeted therapeutic drugs to cancer cells via macropinocytosis [9,84,85]; inducing methuosis in cancer cells with the excessive activation of macropinocytosis [86,87,88]; and inhibiting macropinocytosis to block nutrient uptake in cancer cells [89,90,91]. Currently, utilizing macropinocytosis to deliver therapeutic drugs or agents is the major strategy being applied in cancer therapy. Macropinocytosis-based anti-cancer therapy is applied in chemo/targeted therapy, immunotherapy, and nucleic acid therapy either by macropinocytic delivery of anti-cancer chemo/targeted drugs, antibodies, or vaccines or nucleic acids into cancer cells, or by the regulation of macropinocytosis using macropinocytic inhibitors or activators to induce nutritional stress or methuosis [92]. 

(1) Macropinocytosis-associated chemo/targeted therapy. Macropinocytosis-associated chemo/targeted therapy includes two aspects: one is the utilization of macropinocytosis to deliver anticancer chemo/targeted drugs or agents into cancer cells to produce anti-cancer effects. The other is to employ macropinocytic inhibitors or activators to inhibit or over-activate macropinocytosis to either induce nutritional stress or methuosis of cancer cells. The chemo/targeted drugs or agents that currently used for cancer therapy by either utilizing macropinocytosis for delivering or inhibiting macropinocytosis are shown in Table 1. 

RAS mutant cancer cells have very active macropinocytosis [9]. Thus, the utilization of the hyper-active macropinocytosis to deliver chemo drugs into cancer cells is an efficient means for cancer therapy. It has been demonstrated that chemo/targeted drugs can bind to albumin and enter KRAS-mutated PDAC cells via macropinocytosis [85,93]. Paclitaxel (PTX) is a chemotherapeutic drug for multiple types of cancer. To facilitate the uptake of PTX by RAS mutant cancer cells, PTX is formulated with albumin nanoparticles [84]. The albumin-nanoparticle-wrapped PTX is internalized through active macropinocytosis, resulting in the inhibition of cancer cells’ growth [84]. With a similar strategy, other chemo drugs or anti-cancer agents, such as doxorubicin, human β-defensin-2 peptide, and triptolide, were used in cancer therapy [9]. Tubeimoside-1 (TBM1), a natural triterpenoid saponin found in the traditional Chinese herbal medicine Bolbostemmatis Rhizoma, is capable of acting as a drug transporter by stimulating macropinocytosis [86]. Furthermore, the β-defensin-2-loaded human serum albumin (DF-HSA) [94], gefitinib-loaded EVs [95], and triptolide prodrug-loaded UPSM (T-UPSM) [96] are all effectively delivered into cancer cells by macropinocytosis for cancer therapy.

Few specific macropinocytic inhibitors have been identified so far for cancer therapy. 5-(N-ethyl-N-propyl) amiloride (EIPA), the inhibitor of Na^+^/H^+^-exchanger 3 (NHE3), is commonly used for inhibiting macropinocytosis [97]. A recent study found that EIPA combined with the proteasomal inhibitor bortezomib packed in pH-responsive polymersomes was able to effectively inhibit the growth of lung cancer A549 cell-xenografted mouse tumors [98]. As shown in Table 1, the vesicular H^+^-ATPase inhibitor Bafilomycin A1 [66,97], the PAK1 inhibitor IPA-3 [22], the actin polymerization inhibitors [31], the PI3K inhibitors [25], the DOCK1 inhibitor TBOPP [99] and the lysosomal inhibitor HCQ [64] are all able to inhibit macropinocytosis and have been used for cancer therapy. The mTORC1/2 inhibitors OSI-027, PP242 [100], and Torin 1 [101,102], the CK2 inhibitor silmitasertib [87,103], an ursolic-acid-derived small molecule (compound **17**) [104], the quinolone derivative Vacquinol-1 [105], Platycodin D (PD) [106], jaspine B (JB) [107], and Epimedokoreanin C (EKC) [108] have been used for anti-cancer therapy via the promotion of macropinosomal vacuolization and by inducing methuosis, thereby killing cancer cells. 

(2) Macropinocytosis-associated immunotherapy. Macropinocytosis-associated immunotherapy either delivers anti-cancer antibodies or vaccines into cancer cells by macropinocytosis or uses immune molecules to specifically target macropinocytosis [109]. Examples of macropinocytosis-associated immunotherapy are listed in Table 1. The Fv-LDP-D3 construct, which comprises the scFv antibody and albumin structural domains, has been shown to promote the efficient internalization of anticancer drugs via macropinocytosis [110]. It was observed that the interaction of CD99 with 0662-mAb induces methuosis by over-activating the IGF-1R/RAS/RAC1 signaling cascade [79]. AGS-16C3F, an antibody-drug conjugate (ADC) against ectonucleotide pyrophosphatase/phosphodiesterase 3 (ENPP3), is currently in development for the treatment of metastatic renal cell carcinoma [111]. AGS-16C3F is internalized in cancer cells via macropinocytosis and thus inhibits cancer cell proliferation [111]. The bacillus Calmette–Guerin (BCG) vaccine for tuberculosis (TB) has been shown to be effective in the treatment of bladder cancer by macropinocytic delivery, which is dependent on RAC1, CDC42, and its effector PAK1 [112]. The mycobacterium tuberculosis vaccine (MTBVAC) is now being developed as a new immunotherapeutic drug for bladder cancer [113]. A biomimetic nanovaccine R837-αOVA-ApoE3-HNP was created for cancer immunotherapy [114]. It contains a poly-(D, l-lactide-co-glycolide) (PLGA) core to encapsulate adjuvant imiquimod (R837), a phospholipid membrane to load antigen peptide (αOVA), and apolipoprotein E3 (ApoE3), to facilitate the internalization of cancer antigens into DCs. It has been demonstrated that the R837-αOVA-ApoE3-HNP mimetic nanovaccine is effectively internalized by the macropinocytosis of dendritic cells, resulting in the activation of antigen-specific T cells and the inhibition of tumor metastasis in a mouse model [114]. 

(3) Macropinocytosis-associated nuclear acid therapy. The anti-cancer agents delivered by macropinocytosis are not limited to chemo drugs or proteins. Anti-cancer RNAs, such as anti-cancer miRNAs and anti-sense RNAs, formulated with exosomes, liposomes, or polymers, can be delivered into cancer cells through macropinocytosis. Macropinocytosis-associated nucleic acid therapy utilizes macropinocytosis to deliver nanoparticle-, lipid-, or polymer-formulated anti-cancer nucleic acids into cancer cells for cancer therapy [115,116] (Table 1). Although nucleic acid therapy has not been developed as much as the other therapies, its potential benefits have been increasingly noted. For instance, exosomes containing specific siRNA or shRNA against oncogenic KRAS-G12D inhibit the growth of pancreatic cancer through macropinocytosis [117]. It has been shown that CXCR4-receptor-stimulated lipoprotein-like nanoparticle-loaded miRNA-34a is internalized by the macropinocytosis of glioma-initiating cells, effectively inhibits their stemness and chemo-resistance, and enhances the survival of mice with glioma-initiating cells [118]. Cancer cells can take up nanostructures carrying activating transcription factor-5 (ATF5) siRNA through macropinocytosis, leading to apoptosis [115]. Furthermore, it was observed that the antisense oligonucleotides of translationally controlled tumor protein (TCTP) with the lipid-modified 5′ end were effectively internalized by the macropinocytosis of castration-resistant prostate cancer cells (CRPCs) [119]. This macropinocytic delivery of TCTP antisense oligonucleotides down-regulated the expression of TCTP, reduced the level of cancer cell viability, and significantly delayed CRPC-xenografted tumor progression in a mouse model [119].

**Table 1:** Therapeutic modalities exploiting macropinocytosis in cancers.

Therapeutic Modalities	Drugs	Mechanisms	Cancers	References
Chemo/targeted therapy	PTX	Drug delivery via macropinocytosis	RAS-driven cancer	[84]
	TBM1	Drug delivery via macropinocytosis	Colon cancer	[86]
	DF-HSA	Drug delivery via macropinocytosis	RAS-driven cancer	[94]
	gefitinib	Drug delivery via macropinocytosis	Lung cancer	[95]
	T-UPSM	Drug delivery via macropinocytosis	PDAC	[96]
	EIPA	Inhibition of NHE3 to influence submembrane pH	RAS-driven cancer	[97,98]
	Bafilomycin A1	Impairment of lysosomal pH	RAS-driven cancer	[66,97]
	IPA-3	Inhibition of actin polymerization	RAS-driven and Wnt-driven cancer	[22]
	cytochalasin D	Inhibition of actin polymerization	RAS-driven cancer	[31]
	wortmannin	Inhibition of PI3K signaling pathway	RAS-driven cancer	[25]
	LY294002	Inhibition of PI3K signaling pathway	RAS-driven cancer	[25]
	TBOPP	Preventing DOCK1 from activating RAC1	RAS-driven cancer	[99]
	HCQ	Blocking lysosomal acidification	Pancreatic cancer	[64]
	OSI-027	Induction of macropinocytosis	Brain cancer, colorectal cancer, cervical cancer, breast cancer, lung cancer	[100]
	PP242	Induction of macropinocytosis	Brain cancer, colorectal cancer, cervical cancer, breast cancer, lung cancer	[100]
	Torin 1	Induction of macropinocytosis	RAS-driven cancer	[101,102]
	silmitasertib	Induction of macropinocytosis	Colorectal cancer, oral squamous cell carcinoma	[87,103]
	compound **17**	Induction of macropinocytosis	Cervical cancer, liver cancer, fibrosarcoma, breast adenocarcinoma, neuroblastoma	[104]
	Vacquinol-1	Induction of methuosis	Glioblastoma	[105]
	PD	Induction of methuosis	A549 lung cancer cell, MCF7 breast cancer cell	[106]
	JB	Induction of methuosis	HGC-27 Stomach cancer cell	[107]
	EKC	Induction of methuosis	NCI-H292 and A549 lung cancer cells	[108]
	MOMIPP	Induction of methuosis	Glioblastoma	[75,80]
Immunotherapy	Fv-LDP-D3	Targeting EGFR	Pancreatic cancer	[110]
	CD99 with 0662mAb	Induction of methuosis	Ewing sarcoma	[79]
	AGS-16C3F	Using macropinocytosis	Metastatic renal cell carcinoma	[111]
	BCG	Using macropinocytosis	Bladder cancer	[22,112]
	MTBVAC	Using macropinocytosis	Bladder cancer	[113]
	R837-αOVA-ApoE3-HNP bionic nanovaccine	Using macropinocytosis	Lung cancer	[114]
Nucleic acid therapy	KRAS-G12D siRNA	Downregulation of KRAS-G12D expression	Pancreatic cancer	[117]
	miRNA-34a	Using macropinocytosis	Glioma	[118]
	TCTP ASOs	Downregulation of TCTP expression	Prostate cancer	[119]
	ATF5 siRNA	Using macropinocytosis	Glioblastoma	[115]

## 8. Conclusions

In summary, as shown in Figure 1, macropinocytosis initiates with the formation of plasma membrane ruffles, followed by the production of macropinocytic cups, which in turn generate early macropinosomes. The early macropinosomes continue to mature into late macropinosomes, which fuse with lysosomes and degrade their cargos. Some of the early macropinosomes are able to recycle back to the plasma membrane. Excessive macropinosomes produced by overactive macropinocytosis may fuse with each other to form large vacuoles, leading to methuosis and killing the cell. Therefore, macropinocytosis is a dynamic process with multiple signaling pathways involved. The central signaling molecules involved in the activation of macropinocytosis are RAS, RAC1, and PI-3 kinases. Many signaling pathways, including growth factors, nutrient stress, hypoxia, and pathogen invasion, activate or regulate macropinocytosis through signaling convergence to the RAS, RAC1, and/or PI-3 kinases.

As an important cellular metabolic process, macropinocytosis is associated with other metabolic process, such as autophagy, for cell survival and growth, particularly for cancer cell survival and growth under nutritional or oxidation stress. RAS, the key regulatory protein of macropinocytosis, is a known oncogenic protein with a high tumorigenic mutation frequency in solid tumors. This has increased interest in macropinocytosis regarding its function in cancer. Many studies have shown that macropinocytosis plays an oncogenic role in cancer cells. Thus, targeting or utilizing macropinocytosis is an important strategy to improve current cancer therapeutic outcomes, especially for RAS mutation-mediated tumors.

## Abbreviations

EVs, extracellular vehicles; DCs, dendritic cells; PAK1, P21 Protein Activated Kinase 1; PI, Phosphatidylinositol; EGFR, epidermal growth factor receptor; PDGF, platelet-derived growth factor receptor; CSF-1R, colony-stimulating factor-1 receptor; PI3K, phosphoinositide 3-kinase; CaSR, calcium-sensing receptor; GPCR, G protein-coupled receptor; DAG, diacylglycerol; PLC, phospholipase C; PKC, protein kinase C; SFK, Src-family kinases; PA, phosphatidic acid; PRs, peripheral ruffles; CDRs, circular dorsal ruffles; PI(3,4,5)P3, Phosphatidylinositol-3,4,5-trisphosphate; TIAM1, T-lymphoma invasion and metastasis 1; DOCK2, dedicator of cytokinesis 2; PI(4,5)P2, Phosphatidylinositol 4,5-bisphosphate; SCAR, suppressor of cAMP receptor; WAVE, WASP family verprolin homologous; WASP, Wiskott–Aldrich syndrome protein; Arp2/3, Actin-Related Proteins2/3; MEF, Mouse Embryo Feeder; PI(3,4)P2, phosphatidylinositol 3,4-bisphosphate; PI4P, phosphatidylinositol 4-phosphate; LY, Lucifer yellow; TPC, two-pore channel; PIKfyve, phosphoinositide kinase, FYVE-type zinc finger containing; ALS2, Amyotrophic lateral sclerosis 2; LRRK, leucine-rich repeat kinase; EHBP1L1, EH domain binding protein 1-like 1; HCQ, Hydroxychloroquine; V-H(+)-ATPase, vesicular H(+)-ATPase; TRPML1, Transient receptor potential mucolipin 1; PTX, paclitaxel; TBM1, tubeimoside-1; T-UPSM, Triptolide prodrug-loaded UPSM; EIPA, 5-(N-ethyl-N-propyl) amiloride; NHE3, Na^+^/H^+^-exchanger 3; PD, Platycodin D; JB, Jaspine B; EKC, Epimedokoreanin C; ADC, antibody-drug conjugate; ENPP3, ectonucleotide pyrophosphatase/phosphodiesterase 3; BCG, Bacille Calmette-Guérin; TB, tuberculosis; MTBVAC, mycobacterium tuberculosis vaccine; ATF5, activating transcription factor-5; TCTP, translationally controlled tumor protein; CRPCs, castration-resistant prostate cancer cells.
